# Metabolomic Profiling Reveals That 5-Hydroxylysine and 1-Methylnicotinamide Are Metabolic Indicators of Keloid Severity

**DOI:** 10.3389/fgene.2021.804248

**Published:** 2022-02-09

**Authors:** Mengjie Shan, Hao Liu, Yan Hao, Kexin Song, Tian Meng, Cheng Feng, Youbin Wang, Yongsheng Huang

**Affiliations:** ^1^ Department of Plastic Surgery, Peking Union Medical College Hospital, Beijing, China; ^2^ Graduate School, Chinese Academy of Medical Sciences and Peking Union Medical College, Beijing, China

**Keywords:** keloid, metabolomic, random forest, PLS-DA, machine learning, neural network model

## Abstract

**Background:** Keloid is a skin fibroproliferative disease with unknown pathogenesis. Metabolomics provides a new perspective for revealing biomarkers related to metabolites and their metabolic mechanisms.

**Method:** Metabolomics and transcriptomics were used for data analysis. Quality control of the data was performed to standardize the data. Principal component analysis (PCA), PLS-DA, OPLS-DA, univariate analysis, CIBERSORT, neural network model, and machine learning correlation analysis were used to calculate differential metabolites. The molecular mechanisms of characteristic metabolites and differentially expressed genes were identified through enrichment analysis and topological analysis.

**Result:** Compared with normal tissue, lipids have a tendency to decrease in keloids, while peptides have a tendency to increase in keloids. Significantly different metabolites between the two groups were identified by random forest analysis, including 1-methylnicotinamide, 4-hydroxyproline, 5-hydroxylysine, and l-prolinamide. The metabolic pathways which play important roles in the pathogenesis of keloids included arachidonic acid metabolism and d-arginine and d-ornithine metabolism. Metabolomic profiling reveals that 5-hydroxylysine and 1-methylnicotinamide are metabolic indicators of keloid severity. The high-risk early warning index for 5-hydroxylysine is 4 × 10^8^-6.3×10^8^ (*p* = 0.0008), and the high-risk predictive index for 1-methylnicotinamide is 0.95 × 10^7^-1.6×10^7^ (*p* = 0.0022).

**Conclusion:** This study was the first to reveal the metabolome profile and transcriptome of keloids. Differential metabolites and metabolic pathways were calculated by machine learning. Metabolomic profiling reveals that 5-hydroxylysine and 1-methylnicotinamide may be metabolic indicators of keloid severity.

## 1 Introduction

Keloid is a pathological fibroproliferative disease (Ogawa, 2017). Its characteristics of invasion and migration to surrounding tissues are similar to the growth pattern of benign tumors, but it does not metastasize (Andrews et al., 2016). Keloids often protrude on the original skin lesion area accompanied by pain and itching, which causes a serious burden on the patient’s quality of life and even affects limb function (Berman et al., 2017). Although there are many methods for the treatment of keloids, due to the unknown pathogenesis and high recurrence rate, safe and effective treatment and prevention protocols have not been identified. Therefore, exploring the pathogenesis of keloids will help find effective biomarkers and therapeutic targets to inhibit or eliminate keloids.

The current research mostly describes the pathogenesis of keloids from the perspectives of genetic susceptibility, immunity, and inflammation (Ogawa, 2017). Metabolomics is a new high-throughput sequencing discipline after genomics, transcriptomics, and proteomics, and it is an important part of reflecting changes in the body (Rinschen et al., 2019). Metabolomics is a subdiscipline of systems biology based on cluster index analysis, using high-throughput detection and data processing as the means and aiming at information modeling and system integration (Bujak et al., 2015). The metabolome refers to all of the low–molecular weight metabolites of a certain organism or cell in a specific physiological period (Bujak et al., 2015). Researchers conduct qualitative and quantitative analyses of metabolites to explore and predict diagnosis- and prognosis-related biomarkers that reflect the disease development status of treatment targets. 5-Hydroxylysine is usually present in collagen in the form of glycosylation (Herbert et al., 2012). 1-Methylnicotinamide is an immune regulatory metabolite in human ovarian cancer (Kilgour et al., 2021). 1-Methylnicotinamide regulates thrombogenesis and inflammatory processes (Chlopicki et al., 2007). The role of these two metabolites in keloid has not been studied. Research on metabolomics based on keloids is still needed. Our research explores the mechanism of metabolites in the development of keloids from the perspective of metabolomics.

## 2 Materials and Methods

### 2.1 Human Samples

This study was approved by the Medical Ethics Committee of Peking Union Medical College Hospital (JS-2907), China. All participants provided written informed consent.

From May 2019 to May 2021, a total of 35 patients were included in this study, including 12 cosmetic patients and 23 keloid patients. Patients were 20–47 years old (17 males, 18 females). The collected specimens were divided into two groups: 20 keloid tissues from keloid patents (group K) were used as the experimental group. Eight normal skin tissues from keloid patients (group N) and 12 healthy tissues from cosmetic patients (group C) served as the control group. Five keloid tissues and five normal skin tissues were obtained from the same patient. The modified Vancouver Scar Scale (mVSS) is used to measure the severity of keloids (Ramadan et al., 2021).

All the sequencing samples were from the chest, and the enrolled patients did not have any systemic diseases or receive other treatments to remove possible factors that interfere with the results of the experiment. Normal skin samples surrounding the keloid tissue are 2–3 mm away and need to be removed together during surgery.

### 2.2 Untargeted Metabolome

In our research, we studied the metabolome of samples using a liquid chromatography–mass spectrometry (LC–MS/MS) method. LC–MS data were obtained by coupling an UltiMate 3000 liquid chromatograph (Thermo Fisher Scientific, United States) and a Q Exactive Plus mass spectrometer (Thermo Fisher Scientific, United States). Detailed parameter information is described in the methods of chromatographic conditions and mass spectrometry conditions. The mass spectrometer works in the full scan mode, with a scan rate of 100–1,000 m/z, and automatically performs MS/MS fragment scans. The obtained raw data were converted into mzXML format through Proteowizard software (v3.0.8789) (Chambers et al., 2012). The XCMS package of R (v3.1.3) was used to perform peak identification, peak filtration, and peak alignment. A data matrix was obtained including the mass-to-charge ratio (m/z), retention time (retention time), and peak area (intensity). The positive ion mode obtained 4,193 precursor molecules; 7,163 precursor molecules were obtained in the negative ion mode, and the data were exported to Excel for subsequent analysis. We analyzed 548 skin metabolites confirmed using MS/MS analysis. An LTQ Orbitrap high-resolution mass spectrometry–liquid chromatography mass spectrometer (Thermo Fisher Scientific) was used for the qualitative verification of 5-hydroxylysine and 1-methylnicotinamide. 1-Methylnicotinamide was verified using N-methylnicotinamide (Beijing Soleibao Technology Co., Ltd., SM9380, China). 5-Hydroxylysine was verified using DL-5-hydroxylysine hydrochloride (Sigma–Aldrich, H0377, United States).

### 2.3 Skin Sample Preparation

Fresh surgical specimens were quickly frozen in liquid nitrogen and stored at −80°C. A total of 63.1–101.9 mg of each sample was accurately weighed in a 2-ml EP tube, and 1 ml tissue extract and three steel beads were added (Thermo, Waltham, United States). The sample was placed into a high-throughput tissue grinder (Xinzhi Biological Technology Co., Ltd., SCIENTZ-48, Ningbo, China) and ground for 60 s at 55 Hz. This operation was repeated twice. Ultrasound was performed at room temperature for 30 min, and the samples were placed on ice for 30 min. The samples were centrifuged at 4°C for 10 min at 12,000 rpm, and then 650 μL of the supernatant from each sample was transferred into another 2-ml centrifuge tube. The samples were concentrated and dried in vacuum (Eppendorf, 5,305, Hamburg, Germany). The samples were dissolved in 200 μL of 2-chlorobenzalanine (4 ppm) (Aladdin, 103616-89-3, Shanghai, China) 50% acetonitrile solution (stored at −20°C), and the supernatant was filtered through a 0.22-μm membrane to obtain the prepared samples. Twenty microliters from each sample was tested and mixed into a QC sample. The sample was used for LC–MS detection.

### 2.4 Chromatographic Condition

Chromatographic separation was accomplished in a Thermo Ultimate 3000 system equipped with an ACQUITY UPLC® HSS T3 (150 × 2.1 mm, 1.8 μm, Waters) column maintained at 40°C. The temperature of the autosampler was 8°C. Gradient elution of analytes was performed with 0.1% formic acid in water (C) and 0.1% formic acid in acetonitrile (D) or 5 mM ammonium formate in water (A) and acetonitrile (B) at a flow rate of 0.25 ml/min. Injection of 2 μL of each sample was performed after equilibration. An increasing linear gradient of solvent B (v/v) was used as follows: 0∼1 min, 2% B/D; 1∼9 min, 2∼50% B/D; 9∼12 min, 50–98% B/D; 12∼13.5 min, 98% B/D; 13.5∼14 min, 98∼2% B/D; and 14∼20 min, 2% D-positive model (14∼17 min, 2% B-negative model) (Zelena et al., 2009).

### 2.5 Mass Spectrometry Conditions

The ESI-MSn experiments were executed on a Thermo Q Exactive Plus mass spectrometer, with spray voltages of 3.5 kV and −2.5 kV in positive and negative modes, respectively. Sheath gas and auxiliary gas were set at 30 and 10 arbitrary units, respectively. The capillary temperature was 325°C. The analyzer scanned over a mass range of m/z 81-1,000 for full scan at a mass resolution of 70,000. Data-dependent acquisition (DDA) MS/MS experiments were performed with HCD scans. The normalized collision energy was 30 eV. Dynamic exclusion was implemented to remove some unnecessary information in MS/MS spectra (Zelena et al., 2009).

### 2.6 Data Preprocessing

Data quality control ensures repeatability and accuracy of metabolomics measurements. As the chromatographic system and mass spectrometer are in direct contact with samples, they are easily polluted with an increase in the number of samples to be analyzed, resulting in signal drift and system measurement error. The quality control samples were used to evaluate the signal drift of the mass spectrometry data during the acquisition process. These drifts can be further identified and corrected by precise algorithms to improve the quality of the data. The QC-RFSC algorithm of the R statTarget package (Bioconductor, version 3.13) was used to correct the signal peak of each sample feature.

### 2.7 Bioinformatics Analysis

These metabolites were annotated using the KEGG database (Kanehisa and Goto, 2000) (https://www.genome.jp/kegg/pathway.html), HMDB database (https://hmdb.ca/metabolites), and LIPIDMaps database (http://www.lipidmaps.org/). PLS-DA is an upgraded version of linear discriminant analysis, which is suitable for omics data where explanatory variables have a large number of collinearity problems (Gromski et al., 2015). Orthogonal partial least squares discriminant analysis is an improvement of PLS-DA. In OPLS-DA, the regression model is built between explanatory variables (metabolite data) and response variables (grouping information) that contain grouping information, and the model filters out information that is not related to grouping (Westerhuis et al., 2010). The random forest algorithm is a combination of bagging and decision trees and is used to calculate important metabolites (Hanko et al., 2021). Data normalization, principal component analysis (PCA), partial least squares discriminant analysis (PLS-DA), orthogonal partial least squares discriminant analysis (OPLS-DA), random forest (RF) analysis, and support vector machine (SVM) analysis were performed with the R package MetaboAnalystR. To make the data close to a normal distribution, the normalization function in the MetaboAnalystR package (with arguments MedianNorm, LogNorm, and AutoNorm) was adopted. We applied univariate analysis (*t*-test) to calculate the statistical significance (*p* value). The metabolites with Value Importance in Projection (VIP) > 1, *p* value < 0.05, and log2 (fold change) > 1 were considered to be differential metabolites. For clustering heatmaps, the data were normalized as z scores and plotted by the Pheatmap package in R language. A volcano plot was used to filter metabolites of interest based on log2 (fold change) and -log10 (*p* value), using the ggplot2 package in R language. The metabolites with a *p* value < 0.05 (*t*-test) were used to conduct an overrepresentation analysis (ORA) or enrichment analysis, and the resulting KEGG pathways with a *p* value < 0.05 (ORA) were considered statistically significantly enriched.

### 2.8 Hematoxylin–Eosin Staining

HE staining labels the nucleus in blue–purple and the cytoplasm in red. HE staining was used to observe the morphology of keloid tissue. First, the tissue sections were dried at room temperature for 20 min. Second, the hematoxylin dye was added for 3 min. Third, 0.5% hydrochloric acid alcohol was added for a few seconds to distinguish the samples. Fourth, eosin was added for 30 min, the slides were dehydrated with gradient alcohol, and then xylene was used to make any unstained samples transparent. Finally, the samples were observed under a stereomicroscope.

### 2.9 CIBERSORT

The CIBERSORT deconvolution algorithm is a machine learning method based on linear support vector regression (SVR), implemented through the R package (version 3.5.3, R Foundation for Statistical Computing, Vienna, Austria). The CIBERSORT deconvolution algorithm is used to transform the gene expression information of the transcriptome into the proportion information of immune cells, and the abundance of immune cells can be obtained through visual analysis of histograms, heatmaps, and violin maps. GSE92566 was downloaded (https://www.ncbi.nlm.nih.gov/geo/query/acc.cgi?acc = GSE92566) from GEO datasets. The threshold was set to *p* < 0.05, |log2 (fold change) |≥1. After GSE92566 was normalized, the gene expression information was converted into immune cell abundance information through the CIBERSORT algorithm of the R package (Chen et al., 2018).

### 2.10 Neural Network Model

The neural network model was built in MATLAB (version 9.2.0.538062, MathWorks, United States) using the BP algorithm (multilayer feedforward neural network model) for fitting. The row data are normalized to distribute between [0, 1]. After normalization, the data were randomly divided into a training set and validation set at a ratio of 3:1. The entire neural network model includes an input layer, a hidden layer, and an output layer. The hidden layer was set to 2. The tansig function was used between the input layer and the hidden layer: the digital S-shaped function was for information transmission, and the hidden layer to the output layer adopt the Purelin function (linear function) for transmission. The number of network training iterations is 9,000, and every 1,000 iterations display the primary error. The target error is 10^−5^, and the learning rate is 0.05 based on experience. The momentum factor mc is 0.9. The model is initialized randomly. The output values of output neurons to patients and healthy controls are set to 1–15 (Esmaelpoor et al., 2020).

## 3 Results

### 3.1 Morphological Characteristics of Group K and Control

**FIGURE 1:**
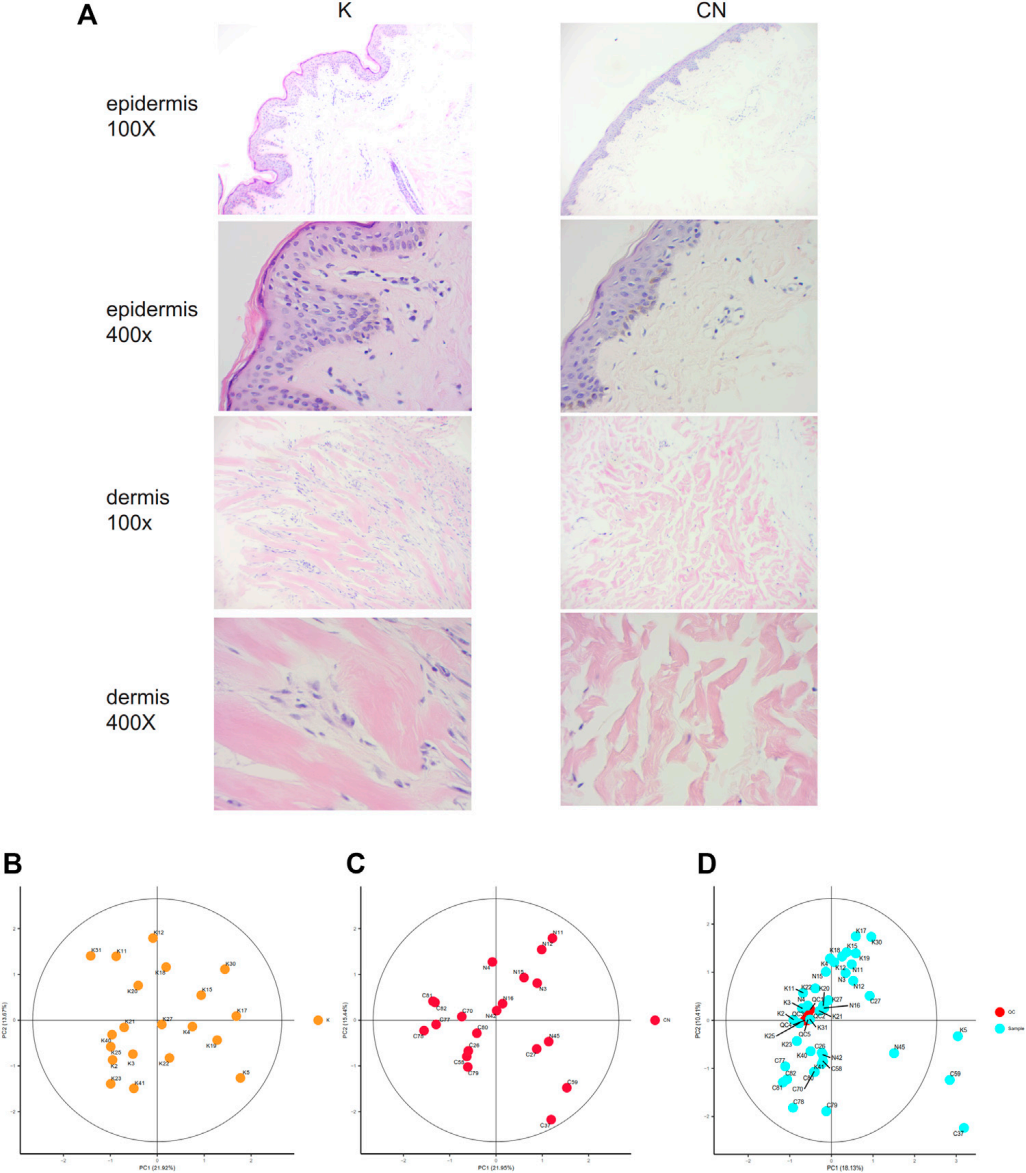
**(A)** Histological images of the K group and control group, 100X. 400X. **(B)** Unsupervised principal component analysis (PCA) of keloid tissues. Each point in the figure represents a sample, and the position of the point in the figure is determined by all the metabolites in the sample. The ellipse in the figure is based on the 95% confidence interval calculated and drawn by Hotelling T2. The sample falling outside the ellipse implies that the point may be an outlier. **(C)** Unsupervised principal component analysis (PCA) of control tissues. **(D)** Calibration signal drift. QC sample points together, proving that the calibration effect is good. The red dots are QC samples after calibration, and the blue dots are test samples.

The collagen fiber bundles in the dermis of keloids were thick, dense, and disorderly arranged and contained many inflammatory cells. The epidermal cells of normal skin were well differentiated and had clear layers. Collagen bundles in the dermis of healthy skin were thin and loosely arranged (Figure 1A). The morphology of normal skin tissue and keloid tissue was quite different. Local lipid metabolism needs further metabolome sequencing.

### 3.2 Quality Assessment of Data

To clarify the profiling of local lipid metabolism in keloids, we used non-targeted metabolome analysis to detect metabolites. We identified 548 metabolites by adjusting the most appropriate experimental conditions. There were 162 metabolites that were significantly different between keloids and normal skin (*p* < 0.05). Using unsupervised principal component analysis (PCA), we modeled each group of samples and then displayed the score plot (Figures 1B,C). This type of intragroup PCA eliminates the interference between groups, allowing us to observe the variation within the group more clearly and find possible outliners. Each point in the figure represents a sample, which is determined by all of the metabolites in the samples. There were no outliers in group K (Figure 1B) or the control (Figure 1C). The quality control (QC) samples were used to evaluate the signal drift of the entire mass spectrum data in the acquisition process, which can be further identified and corrected by accurate algorithms to improve the quality of the data. The QC samples are the samples obtained after all samples are mixed in equal amounts. During the signal data acquisition process, QC samples were inserted at the beginning, end, and middle positions to record the signal drift. There was no signal drift, and the signal strength of QC samples remained unchanged during the data acquisition process (Figure 1D). After the signal drift was corrected, the QC sampling points in the PCA diagram were clustered together, demonstrating that the correction effect was good.

### 3.3 Metabolite Standardization

**FIGURE 2:**
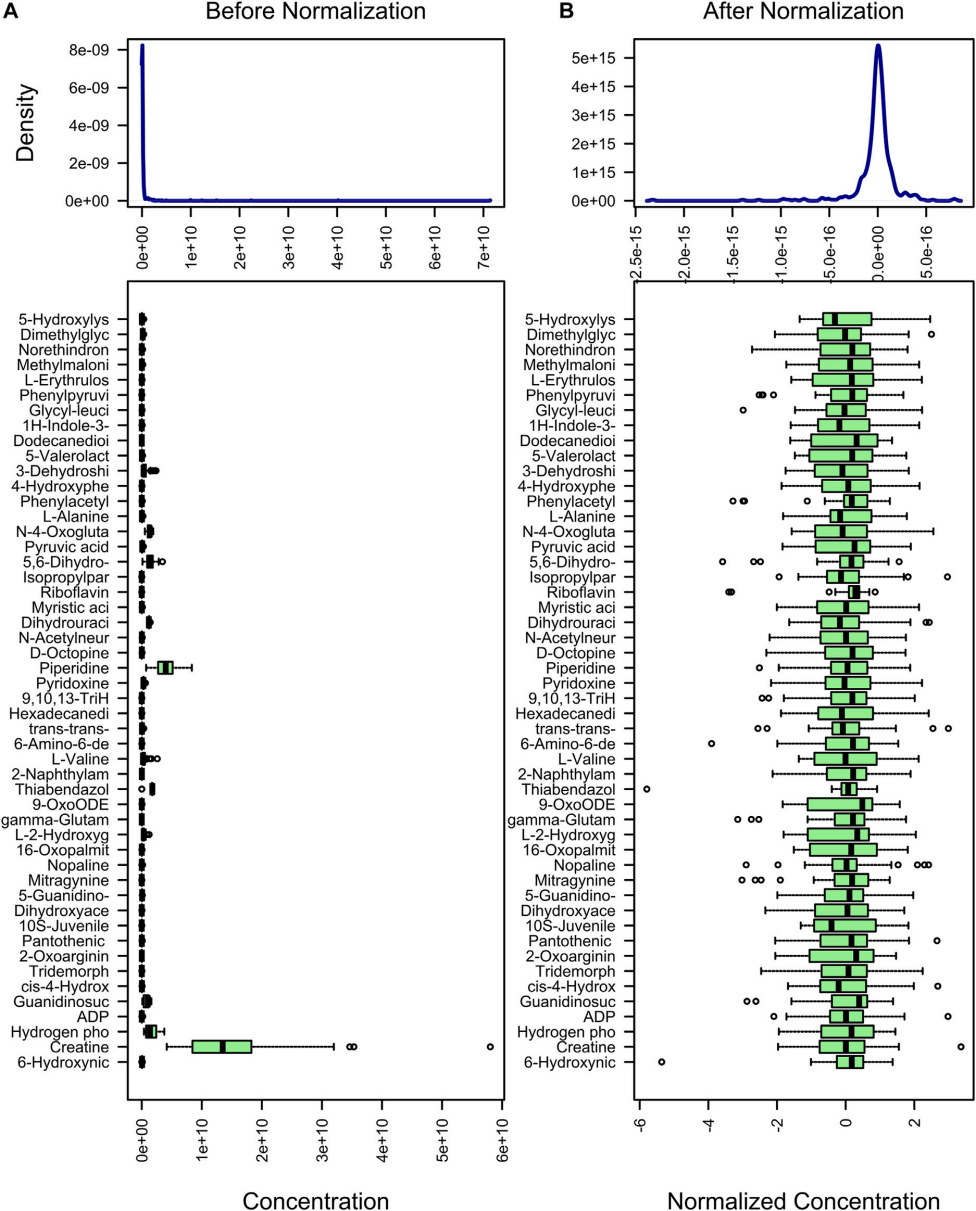
Standardization of metabolites. The content distribution is represented by a box plot, which corresponds to outliers, minimum, lower quartile, median, upper quartile, maximum, and outliers from left to right. The picture on the left is the distribution before normalization, and the picture on the right is the distribution after normalization.

The purpose of metabolite standardization is to make the mean and standard deviation of all metabolites at the same level. For analyses such as PLS-DA, OPLS-DA, and machine learning, if metabolites are not standardized, the importance of metabolites with high mean and standard deviation will tend to be higher than the importance of metabolites with low mean and standard deviation. To accurately analyze metabolites with large differences between groups, metabolite standardization is required. Before standardization, the median and upper and lower quartiles of metabolite content were uneven, and the difference was large (Figure 2A). However, after standardization, they were basically at the same level (Figure 2B). In addition, difference comparison methods such as the *t*-test and ANOVA require that the metabolite content obeys a normal distribution. Therefore, we used log transformation to make the metabolite content distribution close to the normal distribution.

### 3.4 Metabolite Profiles in Keloids Identified by Non-Targeted Metabolomics

**FIGURE 3:**
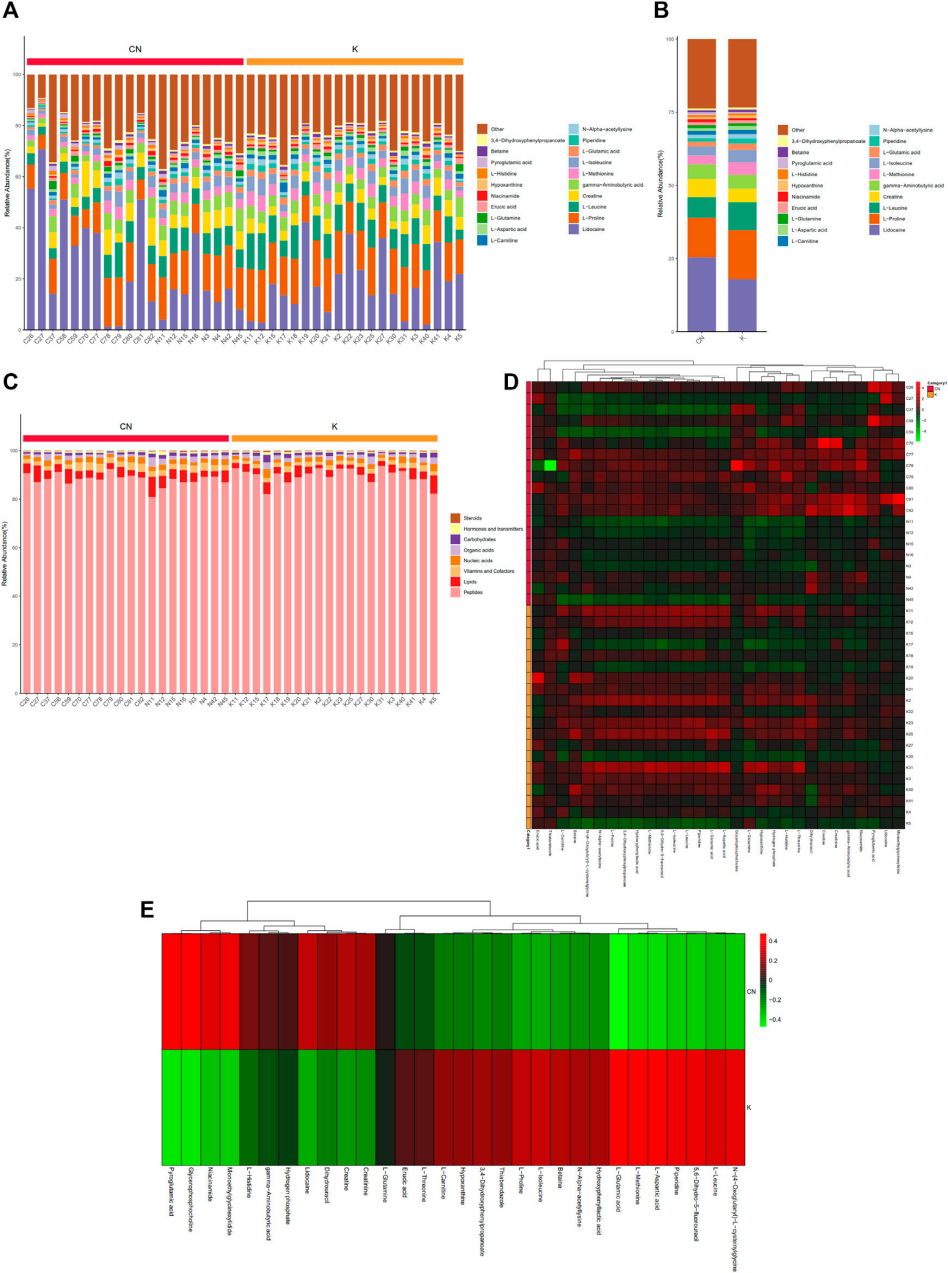
Metabolite content statistics. **(A)** The abscissa is the sample name, sorted according to the grouping order, and different grouped samples are marked with different colors. The ordinate represents the percentage content of each metabolite, and the order of the columns corresponding to the metabolites from top to bottom is consistent with the legend. **(B) (C)** The vertical axis is the sample name information and includes grouping information. The horizontal axis is metabolites. The cluster tree at the top of the figure is the similarity clustering of the metabolite distribution in each sample, the middle heatmap is the metabolite content heatmap, and the relationship between color and metabolite content (Z score) is shown in the upper right scale ruler. **(D)** The expression of metabolites between the two groups.

To calculate the percentage of the content of each metabolite in each sample, a stacked histogram was used, as in Figure 3A, which can intuitively compare the differences in the composition and structure of metabolites between groups. Figure 3A shows the top 20 metabolites, including lidocaine, L-proline, L-leucine, creatine, gamma-aminobutyric acid, and L-methionine. The differences in metabolites between the two groups can be more intuitively realized by combining the samples between the two groups (Supplementary Figure S1A-1B). Creatine, L-leucine, L-proline, and lidocaine seemed to be different between the two groups (Supplementary Figure S1B). The remaining metabolites, more than 20, were included in other groups. We annotated all metabolites with the KEGG database br08001 to obtain the biological roles played by the metabolites, count the percentage content of each biological role, and draw a percentage content stacked column chart, as shown in Figure 3B. Compared with normal tissue, lipids have a tendency to decrease in keloids, while peptides have a tendency to increase in keloids (Supplementary Figure S1). Skin metabolites in keloids play various roles, including peptides, lipids, vitamins and cofactors, nucleic acids, organic acids, carbohydrates, hormones and transmitters, and steroids. Metabolites of concern were selected (the top 30 metabolites were selected by default), and the cluster was conducted according to the metabolite composition of the samples (Figure 3C). N-(4-Oxoglutaryl)-L-cysteinylglycine, L-leucine, 5,6-dihydro-5-fluorouracil, piperidine, L-aspartic acid, L-methionine, and L-glutamic acid were upregulated in group K. Pyroglutamic acid, glycerophosphocholine, niacinamide, monoethylglycinexylidide, and L-histidine were downregulated in group K (Figure 3D). The expression of these metabolites was reversed in the control group.

### 3.5 A PLS-DA Model Was Established to Analyze the Metabolites of Keloids

#### 3.5.1 PLS-DA Looks for Factors That Can Distinguish the Grouping of Samples to the Greatest Extent

**FIGURE 4:**
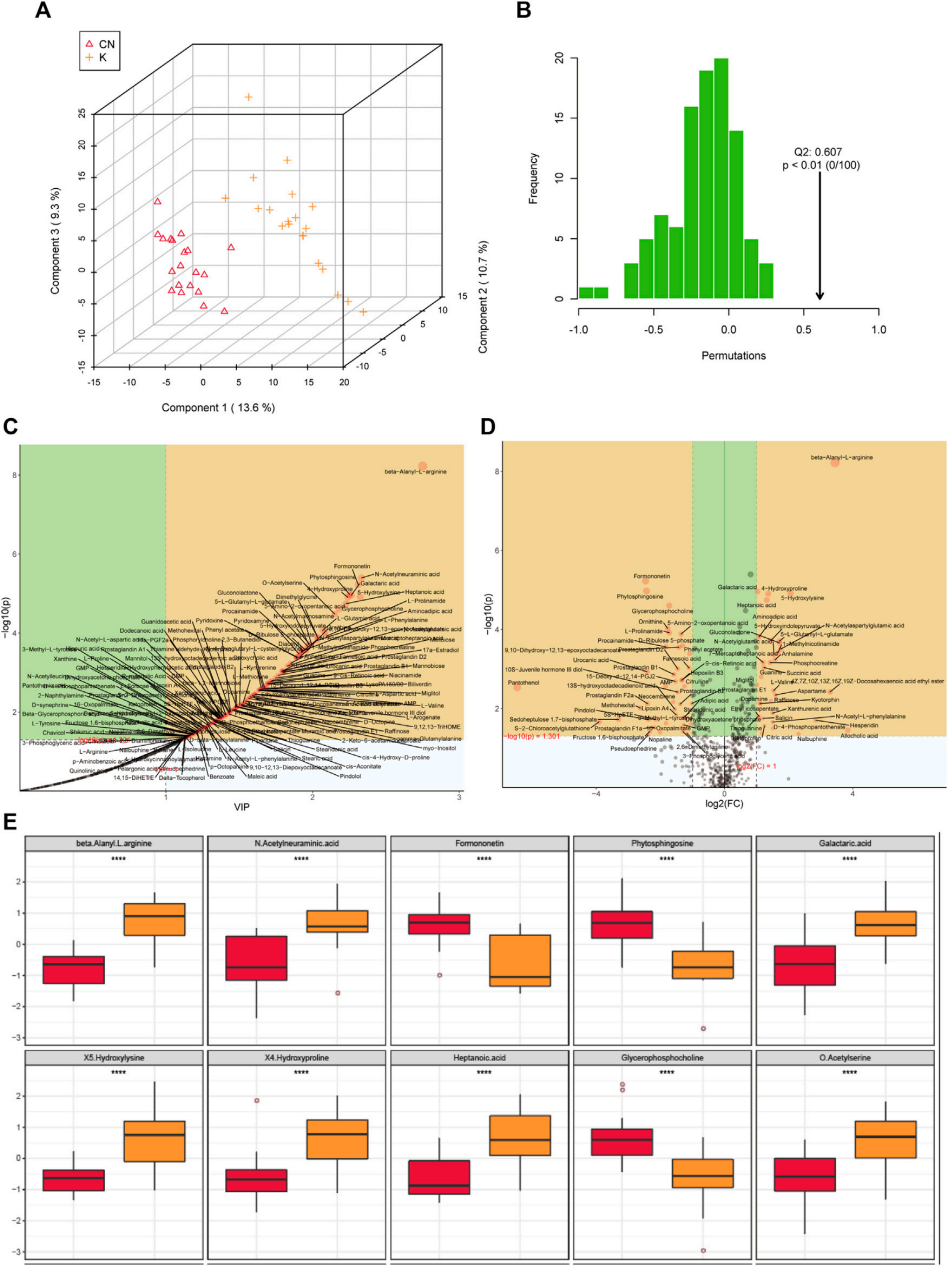
**(A)** Each point corresponds to a sample, and the horizontal and vertical coordinates are the values of the two factors with the best discrimination effect. Different groups are marked with different colors, and the area marked by the ellipse is the 95% confidence area of the sample point. **(B)** The abscissa represents the distribution interval of the replacement test statistic (model prediction accuracy), the ordinate is the frequency of the test statistic in the interval during the replacement process, and the position pointed to by the arrow is the observed test statistic value. If this value is far from the random distribution, the model distinguishing effect is not random, and the model distinguishing effect is significant. **(C)** PLS-DA metabolite importance map. In the figure, each point represents a metabolite, the abscissa is VIP (value importance in projection), and the ordinate is the *p* value after FDR correction (log10 conversion). **(D)** Multiple change volcano map. Each point represents a metabolite, the abscissa is the multiple of change, and the ordinate is the *p* value of the *t*-test. The larger the multiple of change, the smaller the *p* value [the higher the log10(p)], and the larger the point. **(E)** Box diagram of metabolite differences. To visually display the differences in metabolites between groups, we made a box diagram of representative differential metabolites with the top ranking (top 25 with small *p* value) obtained from one-dimensional statistical analysis. *, **, *** correspond to *p* < 0.05, *p* < 0.01, *p* < 0.001.

**FIGURE 5:**
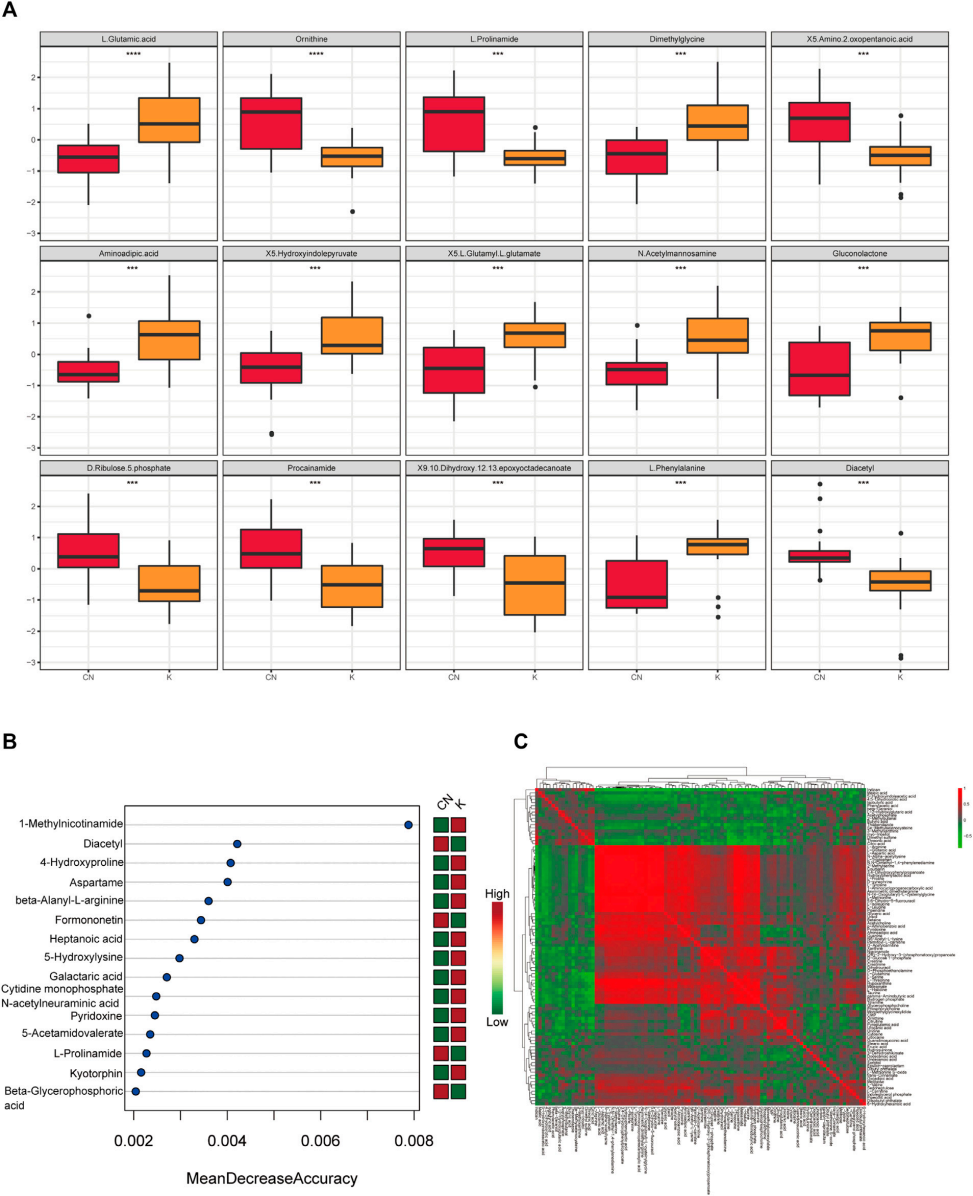
**(A)** Box diagram of metabolite differences. To visually display the differences in metabolites between groups, we made a box diagram of representative differential metabolites with the top ranking (top 25 with small *p* value) obtained from one-dimensional statistical analysis. *, **, *** correspond to *p* < 0.05, *p* < 0.01, *p* < 0.001. **(B)** The 15 most important metabolites in random forests. The abscissa of the left figure is “Mean Decrease Accuracy,” which measures the importance of a metabolite in a random forest; the right figure is a heatmap of the content of metabolites in two groups. **(C)** Pearson correlation heatmap. Correlation coefficient matrix of the top 100 metabolites in total content. The correlation coefficient is represented by color: red is positively correlated, and green is negatively correlated.

Factors can be understood as the weighted sum of all metabolites. Discriminant analysis encodes the discontinuous categorical variable to be predicted as a latent variable, and the latent variable is continuous so that regression can be established between the explanatory variable and the latent variable, which can be solved by least squares regression. As shown in Figure 4A, we used the two factors with the best distinguishing effects to draw a scatter plot. The point clouds of samples in different groups are distributed in different areas, meaning that the PLS-DA model has a good discrimination effect. There were significantly different metabolites in the two groups. In OPLS-DA’s permutation test, we use Q2 as the test statistic and use the permutation method to obtain the random distribution of Q2. As shown in Figure 4B, the actual observation Q2 indicated by the arrow is on the right side of the random distribution (the observed value is greater than the random value), meaning that Q2 is not random but significant, and the predictive ability of the model is significant, that is, there exist significantly different metabolites between the two groups (Figure 4B). The PLS-DA model was used to calculate the importance of metabolites (Figure 4C). Univariate analysis is used to measure how much metabolites differ between groups to assess how much metabolite changes will affect the organism. We measured the magnitude of this change by calculating the fold change (FC) of the metabolite change, combined with the *p* value, to screen some key metabolites. The change multiple of up is positive, and the multiple of down is negative. As shown in Figure 4D, the threshold of the *p* value was less than 0.05, and an absolute value of the fold change greater than 2 was defined as the yellow area. These metabolites had significant differences between groups (Figure 4D) and had large changes, which should be considered in further research. To visually show the difference in metabolites between groups, representative different metabolites were displayed in box diagrams (Figure 4E, Figure 5A, *p* < 0.001).

### 3.6 Machine Learning of Metabolite of Keloids

The random forest algorithm was used to calculate different metabolites in the two groups. The random forest algorithm is a combination of bagging and decision trees. In random forest analysis, we used “Mean Decrease Accuracy” to measure the importance of a metabolite in the random forest to distinguish groups. Changing the value of a metabolite into a random number, the degree of reduction in the accuracy of random forest prediction is “Mean Decrease Accuracy.” The greater the value, the greater the importance of metabolites in the random forest. Figure 5B shows the 15 most important metabolites in random forests, including 1-methylnicotinamide, diacetyl, 4-hydroxyproline, beta-alanyl-l-arginine, heptanoic acid, 5-hydroxylysine, galactaric acid, cytidine monophosphate N-acetylneuraminic acid, l-prolinamide, and beta-glycerophosphoric acid (Table 1). Figure 5C shows the cluster correlation heatmap of the two groups. Correlation coefficient matrix of the top 100 metabolites in total content. The correlation coefficient is indicated by color: red represents a positive correlation, and green represents a negative correlation.

**TABLE 1:** Random forests of metabolites.

Metabolite	Mean decrease accuracy	Mean decrease gini	t.stat	p value
1-Methylnicotinamide	0.008	0.318	−3.958	−3.958
Diacetyl	0.004	0.326	3.990	<0.001
4-Hydroxyproline	0.004	0.191	−5.028	<0.001
Aspartame	0.004	0.152	−3.153	0.004
Beta-alanyl-l-arginine	0.004	0.216	−7.585	<0.001
Formononetin	0.003	0.185	5.301	<0.001
Heptanoic acid	0.003	0.127	4.981	<0.001
5-Hydroxylysine	0.003	0.122	5.355	<0.001
Galactaric acid	0.003	0.143	−5.119	<0.001
Cytidine monophosphate N-acetylneuraminic acid	<0.001	0.029	1.104	0.28
Pyridoxine	0.002	0.124	−3.897	<0.001
5-Acetamidovalerate	0.002	0.1	−3.13	0.004
l-Prolinamide	0.002	0.109	4.51	<0.001
Kyotorphin	0.002	0.076	−2.837	0.007
Beta-glycerophosphoric acid	0.001	0.049	2.185	0.037

### 3.7 Metabolic Pathway Enrichment Analysis of Keloids

**FIGURE 6:**
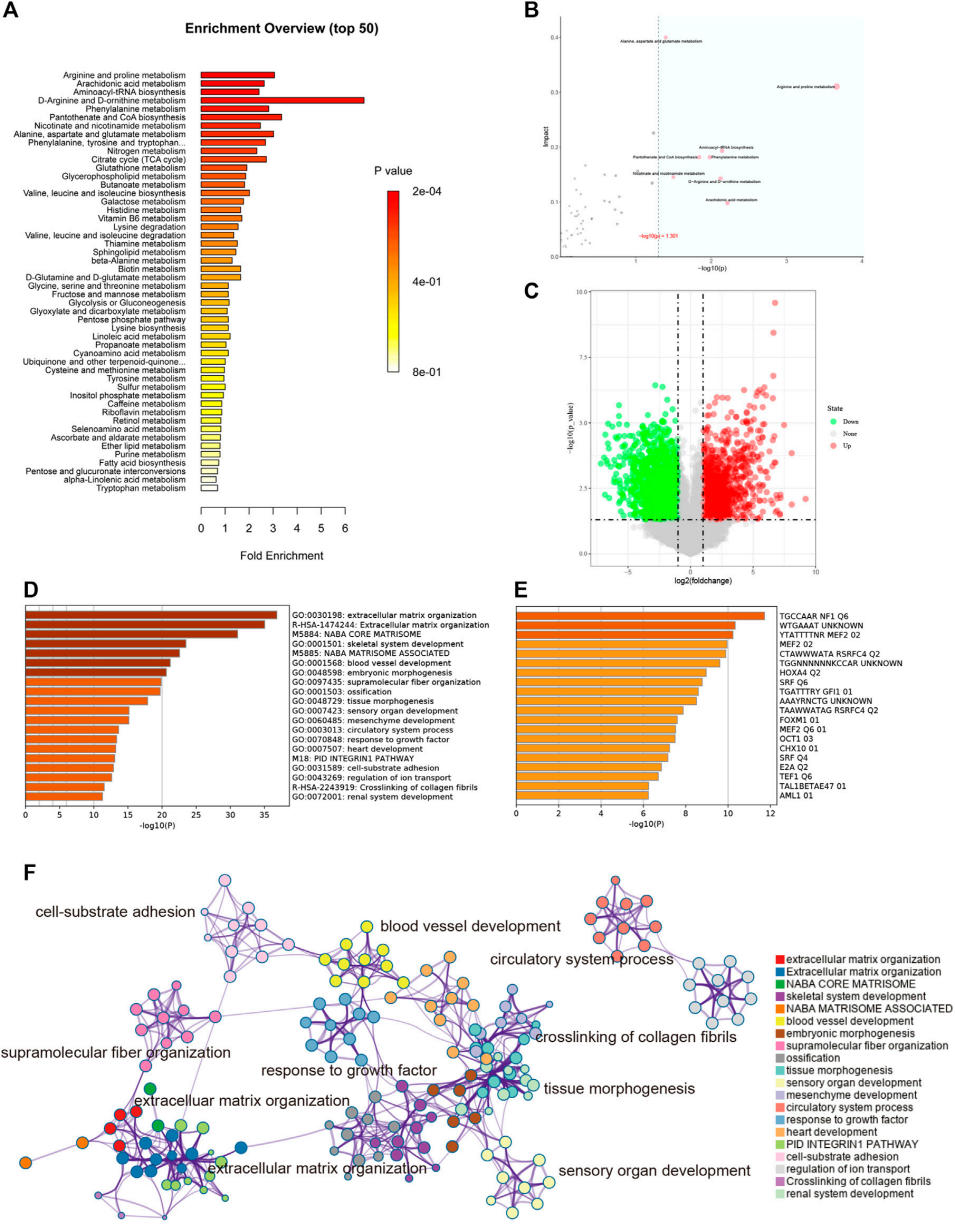
**(A)** Enrichment analysis. The abscissa is the enrichment factor, which is the number of observed metabolites/theoretical metabolites in the metabolic pathway. The size of the *p* value is expressed by color: the darker the color, the smaller the *p* value. **(B)** Topological analysis. The abscissa is the *p* value, and the blue area is significant (*p* < 0.05); the ordinate is the topological analysis impact. **(C)** The volcano plot illustrates the differentially expressed genes between control and keloid tissues after analysis of the GSE92566 dataset with GEO2R. **(D)** GO enriched terms are shown, and accumulative hypergeometric *p* values and enrichment factors were calculated and used for filtering. The remaining significant terms were then hierarchically clustered into a tree based on kappa-statistical similarities among their gene memberships. Then, a kappa score of 0.3 was applied as the threshold to cast the tree into term clusters. **(E)** Transcription factor enriched terms. The darker the color, the higher the enrichment score. **(F)** Metascape analysis. We selected a subset of representative terms from this cluster and converted them into a network layout. More specifically, each term is represented by a circle node, where its size is proportional to the number of input genes falling into that term, and its color represents its cluster identity (i.e., nodes of the same color belong to the same cluster). Terms with a similarity score >0.3 are linked by an edge (the thickness of the edge represents the similarity score). The network was visualized with Cytoscape (v3.1.2) with a “force-directed” layout and with edges bundled for clarity. One term from each cluster is selected to have its term description labeled.

According to the metabolites with significant differences between groups (*p* < 0.05), the biological pathways that play a key role in the pathogenesis of keloids were identified, and the basic molecular mechanism was revealed. Through the KEGG enrichment analysis of metabolic pathways, whether the metabolites of interest are significantly enriched in these metabolic pathways can be assessed. Figure 6A shows the metabolic pathways with significant enrichment of differential metabolites, which may play a role in the occurrence and development of keloids.

Topological analysis can calculate the effect of the metabolite of interest in the metabolic pathway (measured by impact). Therefore, we combined topological analysis and enrichment analysis to determine whether a metabolic pathway plays a key role in the biological process of keloids. The metabolic pathways in the blue area in Figure 6B are significant metabolic pathways in the enrichment analysis, including arachidonic acid metabolism; d-arginine and d-ornithine metabolism; pantothenate and CoA biosynthesis; alanine, aspartate, and glutamate metabolism; arginine and proline metabolism; aminoacyl−tRNA biosynthesis; phenylalanine metabolism; and nicotinate and nicotinamide metabolism (Table 2).

**TABLE 2:** Pathway enrichment of keloid.

Description	Raw p	FDR	Impact	Fold enrichment
Arginine and proline metabolism	0.00021735	0.017388	0.31068	3.0555
Arachidonic acid metabolism	0.006079	0.15052	0.09836	2.6271
Aminoacyl-tRNA biosynthesis	0.00718	0.15052	0.19356	2.413
d-Arginine and d-ornithine metabolism	0.007526	0.15052	0.14286	6.7867
Phenylalanine metabolism	0.010442	0.16707	0.18181	2.8152
Pantothenate and CoA biosynthesis	0.014411	0.19215	0.18181	3.3514
Nicotinate and nicotinamide metabolism	0.031723	0.36255	0.14544	2.4679
Alanine, aspartate, and glutamate metabolism	0.039998	0.39998	0.4	3.0163

### 3.8 Gene Expression Profiles of Keloids

**FIGURE 7:**
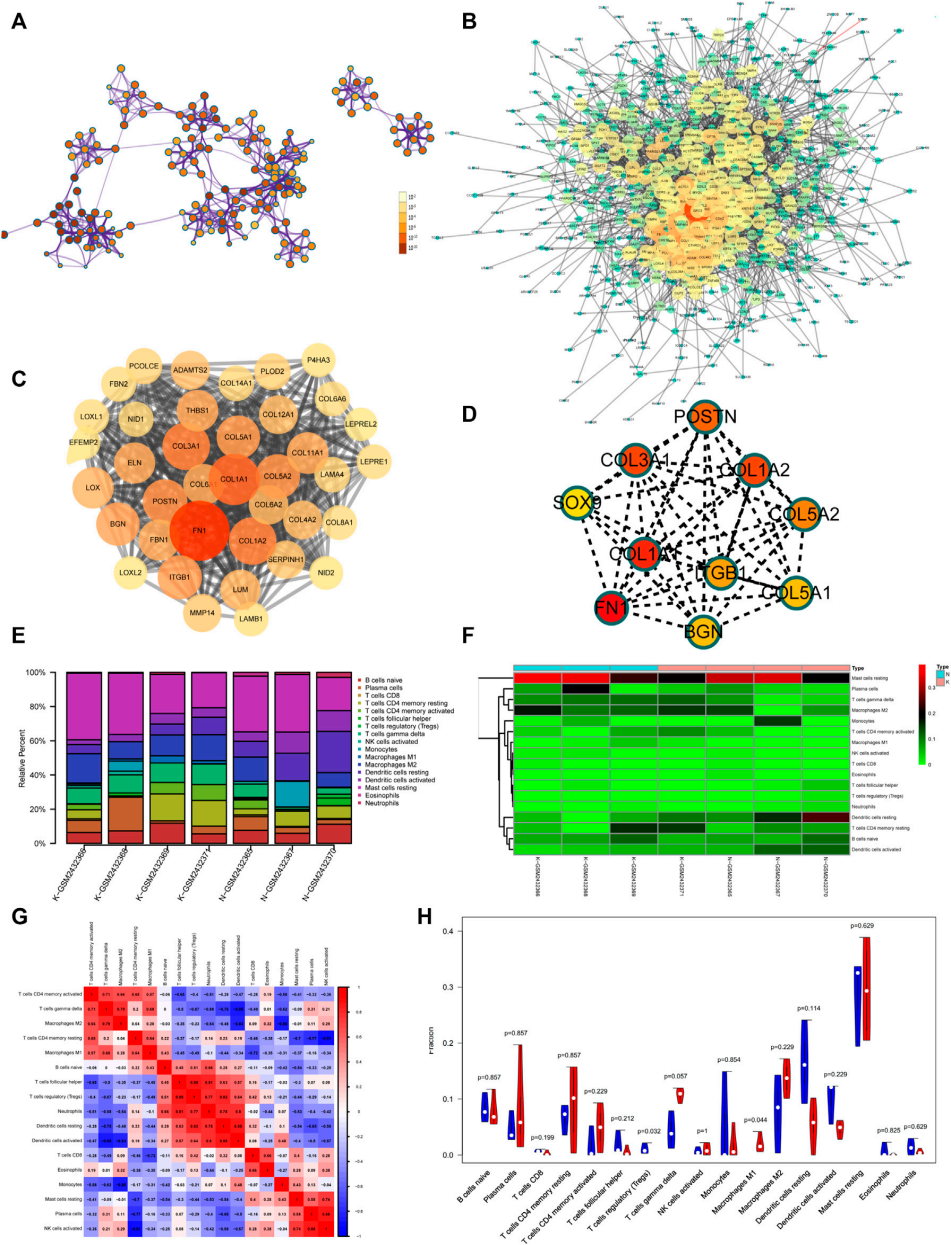
**(A)** Same enrichment network has its nodes colored by *p* value, as shown in the legend. The darker the color, the more statistically significant the node is (see legend for *p* value ranges). **(B)** The protein–protein interaction (PPI) network of differentially expressed genes of GSE92566. **(C)** The MCODE algorithm was used to screen the differentially expressed genes and obtain the important genes of pathogenesis using GSE92566. **(D)** The CytoHubba algorithm was also used to screen the imported genes of GSE92566. **(E)** Fractions of immune cells between control and keloid tissue in the GEO database (GSE92566). **(F)** Heatmap of immune cells between control and keloid tissue in the GEO database (GSE92566). **(G)** A correlational heatmap of immune cells of keloids. **(H)** A violin plot of immune cells found in control and keloid tissue according to the GEO database (GSE92566); blue denotes normal tissue and red denotes keloid tissue.

To better understand the pathogenesis of keloids from multiple angles, GSE92566 was used to reveal changes in gene expression profiles. The threshold was set to *p* < 0.05, |log2 (fold change) |≥1. In total, 3,370 different genes were screened, as shown in Figure 6C. These differentially expressed genes were enriched and related to extracellular matrix organization, NABA CORE MATRISOME, and blood vessel development (Figure 6D). The enrichment results of transcription factors are shown in Figure 6E, including NF1, MEF2, and RSRFC4. Metascape analysis (Zhou Y. et al., 2019) is shown in Figure 6F, including cell–substrate adhesion, blood vessel development, response to growth factor, cross-linking of collagen fibrils, and supramolecular fiber organization. The same enrichment network has its nodes colored by *p* value, as shown in Figure 7A. Differentially expressed genes between keloids and normal tissues were identified by protein–protein interaction (PPI) analysis with complex interactions (Figure 7B). The MCODE algorithm (Zhou Y. et al., 2019) was used to screen the differentially expressed genes and obtain the important genes of pathogenesis (Figure 7C). The CytoHubba algorithm was also used to screen the important genes during keloid formation, including POSTN, COL3A1, COL1A2, SOX9, COL5A2, COL1A1, ITGB1, COL5A1, FN1, and BGN (Figure 7D). The hub genes of COL1A, COL1A2, COL5A2, and COL3A1 indicate that genes related to collagen synthesis play a vital role in the pathogenesis of keloids.

### 3.9 Immune Microenvironment of Keloids

Keloid tissue has a high collagen content, making it difficult to be separated from immune cells in the local immune microenvironment. To better study the local immune microenvironment of keloids, the CIBERSORT algorithm was used (Xiong et al., 2018; Zhou R. et al., 2019). According to the biomarkers of gene expression profile, immune cells are typed to obtain cell abundance information (Xiong et al., 2018; Zhou R. et al., 2019). The histogram and heatmap showed that the information about the proportion of immune cells in group K and group N was uneven, and the local immune microenvironment was different (Figures 7E,F). The relationship between immune cells is shown in Figure 7G. M2 macrophage and gamma delta T-cell proportion synergistically increased (Figure 7G). Treg and resting dendritic cell proportion synergistically increased (Figure 7G). The proportion of Tregs in groups K and N was significantly different (*p* = 0.032). The proportion of Tregs, which plays an important role in immune regulation, was lower than that in normal skin tissue, which may contribute to the vigorous growth of keloid tissue (Figure 7H).

### 3.10 5-Hydroxylysine and 1-Methylnicotinamide are Related to the Severity of Keloids

**FIGURE 8:**
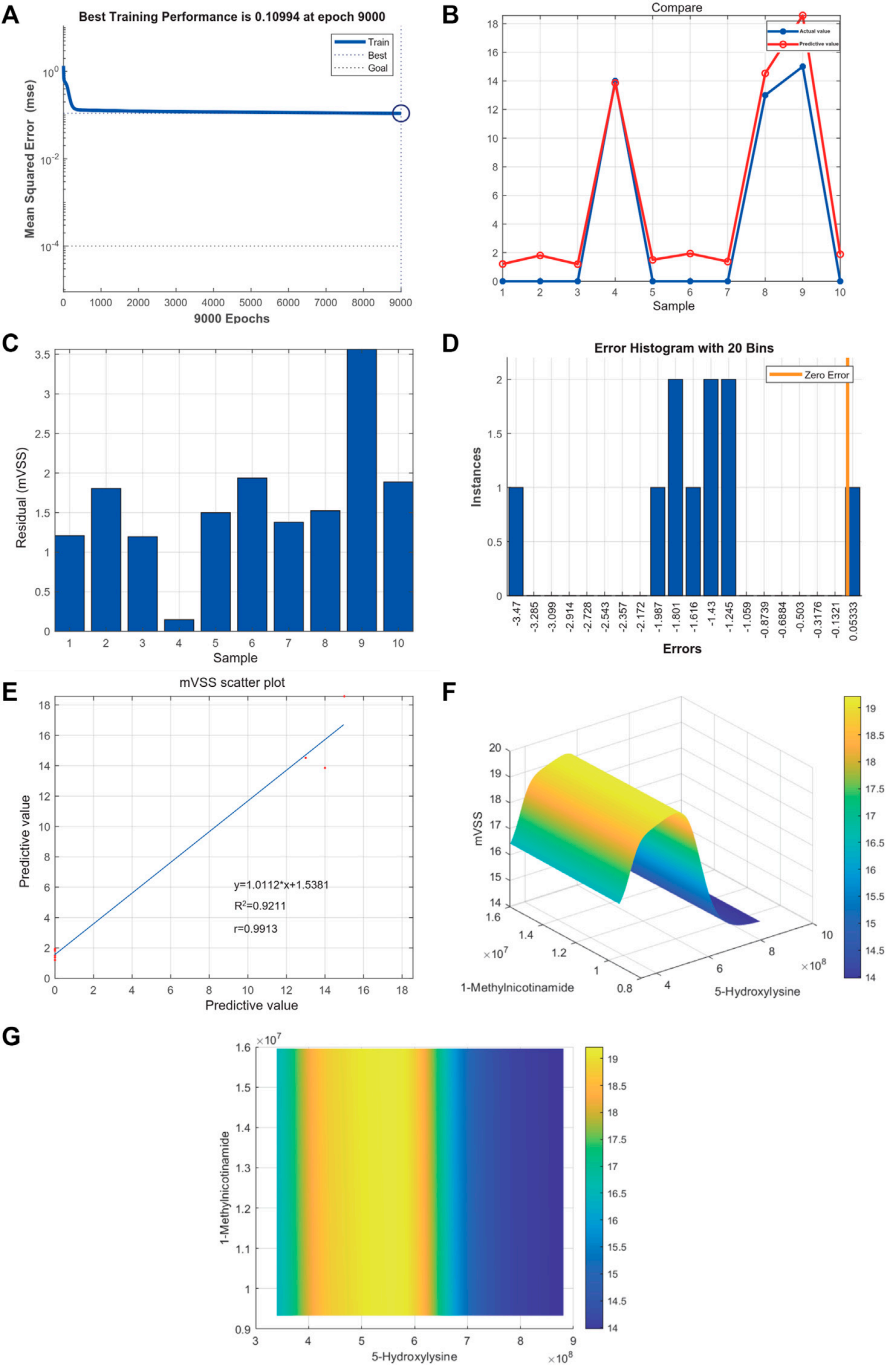
**(A)** Neural network model for the prediction of keloid severity. **(A)** The best training performance was 0.10994 at epoch 9,000. **(B)** The predictive value of the data was verified against the actual value. **(C)** Absolute error diagram between the predicted value and the actual value of the data. **(D)** Error distribution map between the predicted value and the actual value of the data. **(E)** Correlation scatter plot of mVSS. y = 1.0112^*^x+1.5381, *R* ^2^ = 0.9211, r = 0.9913. **(F,G)** High-risk warning range of 5-hydroxylysine and 1-methylnicotinamide at the planform and three-dimensional levels.

**FIGURE 9:**
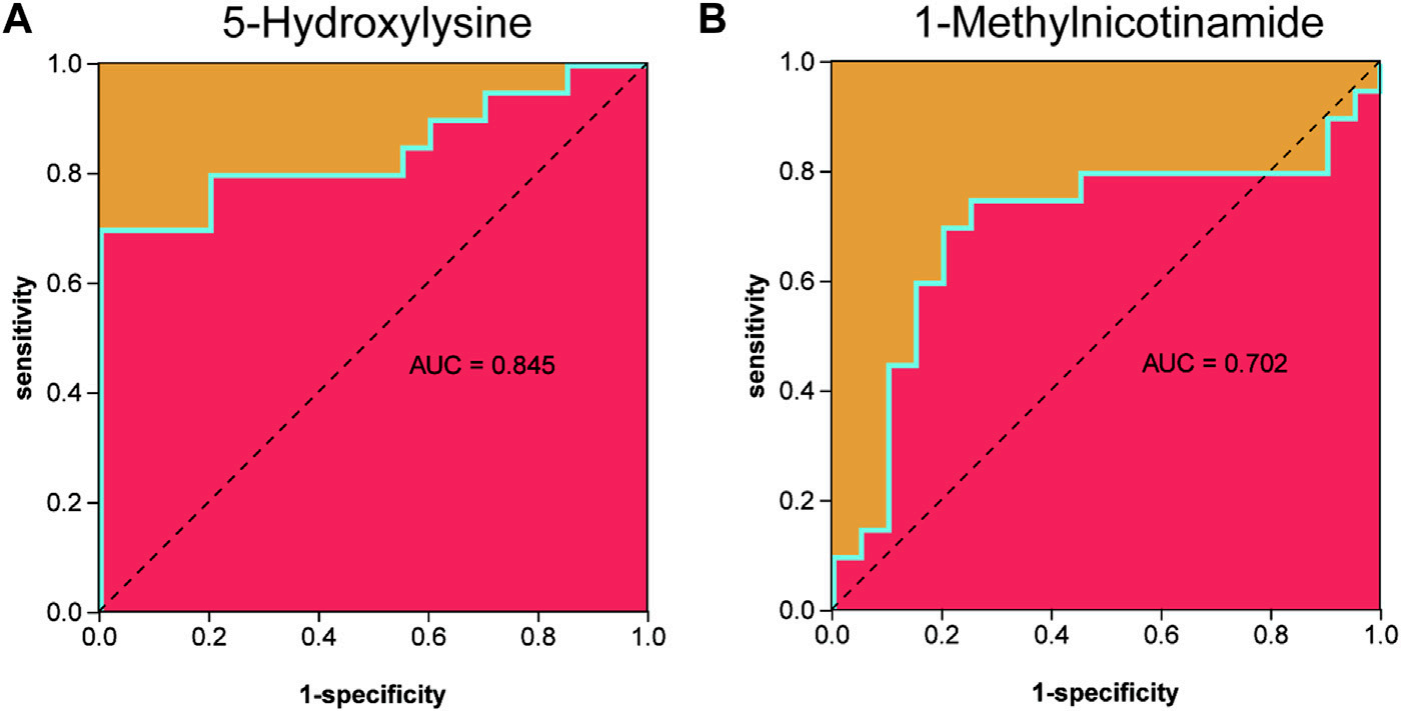
**(A)** Receiver operator characteristic curve indicated that the expression level of 5-hydroxylysine could sensitively and specifically predict keloids. **(B)** The receiver operator characteristic curve indicated that the expression level of 1-methylnicotinamide could sensitively and specifically predict keloids.

Metabolites were easier to obtain in the clinic than transcriptome information, for example, using microneedle punch, tape strip, macroduct sweat collector, and suction chamber (Elpa et al., 2021). Exploring metabolites related to the pathogenesis of keloids can help doctors monitor the development of the disease at any time and provide effective biomarkers for diagnosis, treatment, and even recurrence. To explore the value of 5-hydroxylysine and 1-methylnicotinamide in clinical applications, we incorporated the metabolome sequencing data of 5-hydroxylysine and 1-methylnicotinamide into the construction of the neural network model as the input layer. The mVSS was used as a common index to measure the severity of keloids in the clinic. mVSS was the output layer. Thirty samples were used as the training set, and 10 samples were used as the validation set. The neural network model was successfully built through training. The best training performance was 0.10994 at epoch 9,000 (Figure 8A). The verification set was used to verify the training effect of the neural network model. The agreement between the predicted value and the actual value proved that the training effect is good (Figures 8B,C). The error diagram also showed that the error is acceptable (Figure 8D). The predicted value was consistent with the actual value, and the correlation coefficient was 0.9211 (Figure 8E). The successful construction of the neural network model demonstrated that the expression of 5-hydroxylysine and 1-methylnicotinamide may be predictors of the severity of keloids (Figure 8F). The high-risk early warning index for 5-hydroxylysine was 4 × 10^8^-6.3×10^8^ (*p* = 0.0008), and the high-risk predictive index for 1-methylnicotinamide was 0.95 × 10^7^-1.6×10^7^ (Figure 8G, *p* = 0.0022). The receiver operator characteristic curve shows that the expression level of 5-hydroxylysine can predict keloids sensitively and specifically (AUC, 0.845; 95% CI, 0.715–0.975, Figures 9A, and 1-methylnicotinamide can also predict keloids (AUC, 0.702; 95% CI, 0.524–0.881, Figure 9B).

1-Methylnicotinamide is formed from nicotinamide N-methyltransferase (NNMT). The overexpression of NNMT is associated with the enhanced proliferation, invasion, and metastasis of various cancers (Eckert et al., 2019; Roberti et al., 2021). The expression of aldehyde oxidase 1 (AOX1) converts 1-methylnicotinamide into 1-methyl-2-pyridone-5-carboxamide (2-PYR) or 1-methyl-4-pyridone-5-carboxamide (Kilgour et al., 2021). AOX1 catalyzes the metabolism of 1-methylnicotinamide (Supplementary Figure S2A). The expression levels of NNMT and AOX1 could be analyzed by GSE92566. We found no significant difference in AOX1 between the two groups (Supplementary Figure S2B), and the expression of NNMT in group K was much higher than that of the control (Supplementary Figure S2C, *p* = 0.0008). The expression of NNMT at the transcription level was consistent with the metabolome sequencing data. This finding indicates that 1-methylnicotinamide may promote the vigorous proliferation and invasion of keloids. 5-Hydroxylysine is one of the raw materials of collagen biosynthesis. 5-Hydroxylysine forms due to collagen biosynthesis or posttranscriptional modification disorders (Wu et al., 2019). The formation of collagen fiber cross-links requires posttranslational modification of procollagen-lysine 2-oxoglutarate 5-dioxygenase 2 (PLOD2). Lysine hydroxylase can be divided into three different subtypes based on different functions: PLOD1, PLOD2, and PLOD3. The expression of PLOD1 (Supplementary Figure S2D, *p* = 0.0118) and PLOD2 (Supplementary Figure S2E, *p* = 0.0023) was significantly increased in group K compared to the control. The expression of PLOD3 was not significantly different between the two groups (Supplementary Figure S2F). The increased expression of PLOD1 and PLOD2 regulates the expression of 5-hydroxylysine in keloid tissue. These results also anastomosed with increased expression of COL3A1, COL1A2, COL1A1, COL5A2, and COL5A1 (Figure 7D). Qualitative verification of 1-methylnicotinamide and 5-hydroxylysine was performed using standard products (Supplementary Figure S3).

## 4 Discussion

Keloid is a pathological fibroproliferative disease (Ogawa, 2017). Although an increasing number of studies on keloids have revealed the influence of genetic and environmental factors on their formation, the etiology of keloids is still unclear. Some studies have shown that metabolism is involved, from a metabolome point of view (Bujak et al., 2015). We used non-targeted LC–MS to identify and quantitatively analyze the collected skin tissue samples, and a total of 548 metabolites were identified. There were 162 metabolites that were significantly different between keloids and normal skin (*p* < 0.05). Quality control and a series of analyses were performed on the data, and several important metabolites were finally calculated through the random forest model.

Lipids are an important part of skin and have pro- and anti-inflammatory effects (Louw, 2007; Judge and Dodd, 2020). However, their role in the pathogenesis of keloids has been neglected. Lipids are mainly used as membrane components and second messengers to form biological membrane structures and stratum corneum and play a certain role in local inflammation and intracellular signal transduction (Huang and Ogawa, 2013). Compared with normal tissue, lipids have a tendency to decrease in keloids, while peptides have a tendency to increase in keloids. The decrease in lipids may be a reason for keloid formation.

Skin metabolites of keloids act as peptides, lipids, vitamins and cofactors, nucleic acids, organic acids, carbohydrates, hormones, transmitters, and steroids. The content of lipids is second only to peptides. Lipids include fats (triglycerides), lipids (phospholipids, sterols), sterols and their esters, phospholipids, and glycolipids. Keloid fibroblasts isolated and cultured *in vitro* have a reduced ability to generate PGE2 and EP2 receptors and increase collagen synthesis (Huang and Ogawa, 2013). The expression of insulin-like growth factor I receptor in keloid fibroblasts is higher than that in normal fibroblasts, which inhibits ceramide-induced apoptosis (Ohtsuru et al., 2000). This fact may be the reason why Fas-mediated signaling molecules in keloids are not converted into ceramides. These studies have shown that lipid metabolism plays an important role in keloid formation.

1-Methylnicotinamide is the amide form of vitamin B3 and the main metabolite of nicotinamide (NA) (Przygodzki et al., 2010). The pyridine compound NA is metabolized by nicotinamide N-methyltransferase (NNMT) to form 1-methylnicotinamide. 1-Methylnicotinamide is an activator of prostacyclin production, which can regulate thrombus formation and inflammatory processes (Chlopicki et al., 2007). 1-Methylnicotinamide can improve the pathological changes in mice induced by free fatty acid binding to albumin, such as inflammation, fibrosis, and necrosis. In a study of liver injury, 1-methylnicotinamide improved liver injury by inhibiting the release of the pro-inflammatory cytokines TNF-α and IL-4 (Chlopicki et al., 2007). The expression of 1-methylnicotinamide in keloids is higher than that in normal tissue. The role of 1-methylnicotinamide in the pathogenesis of keloids has not been studied.

4-Hydroxyproline is a non-essential amino acid that is found in collagen and a few other extracellular animal proteins. Hydroxyproline is an important component of the main structural protein of collagen, which affects the stability and synthesis of collagen (Li and Wu, 2018; Tang et al., 2020). Abnormal hydroxyproline can cause defects in collagen synthesis, such as rupture of tendon connective tissue (Srivastava et al., 2016). Hydroxyproline levels reflect collagen metabolism and are significantly higher in keloid tissue. Keloid fibroblasts are similar in appearance and morphology to normal skin fibroblasts, but the hydroxyproline content and collagen production are significantly higher than those of normal skin fibroblasts (Srivastava et al., 2016).

5-Hydroxylysine is usually present in collagen in the form of glycosylation and is an important synthetic target (Herbert et al., 2012). Collagen contains a peptide sequence with a repeating triplet Gly-X-Y, where Y is usually a proline or lysine residue. During collagen synthesis, certain proline and lysine residues undergo hydroxylation and glycosylation, causing the peptide chain to fold into an alpha-helix. The collagen molecule is a triple helix structure. The formation of the triple helix requires repetition of the Gly-Xaa-Yaa sequence (Herbert et al., 2012). Gly-Pro-Hyp is the most common triplet (10.5–22%) in collagen. The preresidue at the Yaa site of the Gly-Xaa Yaa sequence is converted by prolyl-4-hydroxylase (Herbert et al., 2012), a non–heme iron enzyme that exists in the endoplasmic reticulum. The enzyme catalyzes the posttranslational and stereoselective hydroxylation of the inactive *γ*-carbon of the Pro residue at the Yaa position of the collagen sequence to form hydroxyproline. Most proline residues at the Yaa position of vertebrate collagen are hydroxyproline. Hydroxylysine plays an important role in stabilizing the triple helix structure of collagen. Abnormal changes or instability of the triple helix structure may cause a variety of diseases (Krane, 2008; Pálfi and Perczel, 2008; Srivastava et al., 2016). The expression of 5-hydroxylysine is higher than that in normal tissue, which may be the reason why collagen production increases and is difficult to diminish. Based on the metabolome data, we found that a single metabolite cannot accurately reflect the severity of keloids. The successful construction of the neural network model demonstrated that the expression of 5-hydroxylysine and 1-methylnicotinamide may be predictors of the severity of keloids. The high-risk early warning index for 5-hydroxylysine is 4 × 10^8^-6.3×10^8^, and the high-risk predictive index for 1-methylnicotinamide is 0.95 × 10^7^-1.6×10^7^. The results of this study may have practical clinical significance because metabolites are easier to obtain from skin tissues than transcriptomes, for example, they can be easily obtained from skin secretions. The expression level of metabolites can more conveniently reflect the development process of keloids and provide a convenient biomarker for early diagnosis, treatment, and prognosis observation.

Interestingly, when the expression of 5-hydroxylysine was the highest, the symptoms of keloids were not the most serious, which may be related to the complex mechanism of the interaction of 1-methylnicotinamide and 5-hydroxylysine and will also be an interesting research direction in the future to explore the underlying molecular reasons. Transcriptome analysis reveals the pathogenesis of keloids from the perspective of gene expression. Collagen accumulation is an important manifestation of the pathogenesis of keloids (Andrews et al., 2016). Transcriptome analysis showed that the core differentially expressed genes related to collagen synthesis play a vital role in the pathogenesis of keloids, including COL1A, COL1A2, COL5A2, and COL3A1. The analysis of the keloid immune microenvironment revealed the infiltration of immune cells around fibroblasts, which directly or indirectly affected the proliferation of fibroblasts (Zhou et al., 2021). The expression of NNMT, AOX1, PLOD1, PLOD2, and PLOD3 at the transcription level is consistent with the metabolome sequencing data (Supplementary Figure S2). The expression of Tregs in groups K and N was significantly different (*p* = 0.032), which may be related to the vigorous growth of keloid tissue. Tregs play a significant role in immunosuppression and self-tolerance. Tregs indirectly inhibit the production of TGF-*β* by reducing the number of macrophages or directly inhibit the production of TGF-*β* by releasing IL-10. Murao et al. (2014) found that the proportion of Tregs in keloids is relatively low, which is related to our study of inhibition. Coculture of Tregs and keloid fibroblasts can reduce the expression of type I collagen and TGF-*β* in keloids.

This study reveals the metabolome characteristics of keloids for the first time and calculates the metabolites with significant differences between the two groups through machine learning, and enrichment analysis further reveals the possible pathogenesis of keloids. The core genes and immune cells of keloid pathogenesis were identified by transcriptome analysis.

1-Methylnicotinamide is produced by NNMT, which is mainly expressed in tumor cells and fibroblasts. NNMT is overexpressed in various cancers and is related to proliferation, invasion, and metastasis (Eckert et al., 2019; Roberti et al., 2021). The expression of NNMT is restricted to fibroblasts and tumor cells (Kilgour et al., 2021). The overexpression of NNMT promotes metabolism in tumor-associated fibroblasts of the ovary, leading to invasion and metastasis of tumors, and is related to poor prognosis (Kilgour et al., 2021). AOX1 is expressed in fibroblasts, and AOX1 is the enzyme responsible for the degradation of 1-methylnicotinamide (Kilgour et al., 2021). There was no significant difference in AOX1 between the two groups (Supplementary Figure S2B), and the expression of NNMT in group K was higher than that of the control (Supplementary Figure S2C *p* = 0.0008), possibly indicating that the overexpression of NNMT may be a factor influencing the vigorous proliferation of keloid fibroblasts. The expression of PLOD1 and PLOD2 was abnormally increased at the transcriptional level, which affected the abnormal metabolism of 5-hydroxylysine, enhanced collagen cross-linking, and increased deposition.

The expression levels of 5-hydroxylysine and 1-methylnicotinamide can more conveniently reflect the development process of keloids and provide convenient biomarkers for early diagnosis, treatment, and prognostic observation. However, these observations still need to be clinically verified and observed on a larger scale. Although promising metabolites were determined through rigorous bioinformatics analysis and the metabolites were qualitatively verified, there was no richer experimental design and no verification of the enrichment of pathway information. We only discovered the laws of the two metabolites in the neural network model and did not conduct in-depth exploration and experimental verification of the interaction of the two metabolites, which is a limitation and will be further studied in future research.

## Data Availability

The data presented in the study are deposited in the iProX repository, accession number IPX0004021000.
