# Supplementary material for: Metabolomic Profiling Reveals That 5-Hydroxylysine and 1-Methylnicotinamide Are Metabolic Indicators of Keloid Severity
Source: Front Genet. 2022 Feb 9;12:804248. doi: 10.3389/fgene.2021.804248 (PMC8864098; doi:10.3389/fgene.2021.804248)
Supplement: Supplementary file 2 [file Table1.docx]

Supplementary table 1. Characteristic of patients

| Patients^a^ | Age of onset (years) | Gender |
| --- | --- | --- |
| C26 | 24 | female |
| C27 | 42 | male |
| C37 | 20 | female |
| C58 | 34 | male |
| C59 | 32 | female |
| C70 | 22 | female |
| C77 | 23 | Female |
| C78 | 31 | male |
| C79 | 30 | male |
| C80 | 32 | Female |
| C81 | 27 | male |
| C82 | 22 | Female |
| K2 | 23 | male |
| K3, N3 | 25 | Female |
| K4, N4 | 28 | male |
| K5 | 42 | male |
| K11, N11 | 39 | Female |
| K12, N12 | 24 | Female |
| K15, N15 | 27 | male |
| K17 | 36 | Female |
| K18 | 38 | male |
| K19 | 40 | male |
| K20 | 30 | Female |
| K21 | 33 | male |
| K22 | 40 | Female |
| K23 | 28 | male |
| K25 | 41 | male |
| K27 | 32 | Female |
| K30 | 28 | Female |
| K31 | 38 | male |
| K40 | 35 | Female |
| K41 | 25 | Female |
| N16 | 33 | Female |
| N42 | 42 | male |
| N45 | 47 | male |

^a^ K, N and C samples were collected from a total of 35 patients.
