# Supplementary figures and images for: Metabolomic Profiling Reveals That 5-Hydroxylysine and 1-Methylnicotinamide Are Metabolic Indicators of Keloid Severity
Source: Front Genet. 2022 Feb 9;12:804248. doi: 10.3389/fgene.2021.804248 (PMC8864098; doi:10.3389/fgene.2021.804248)

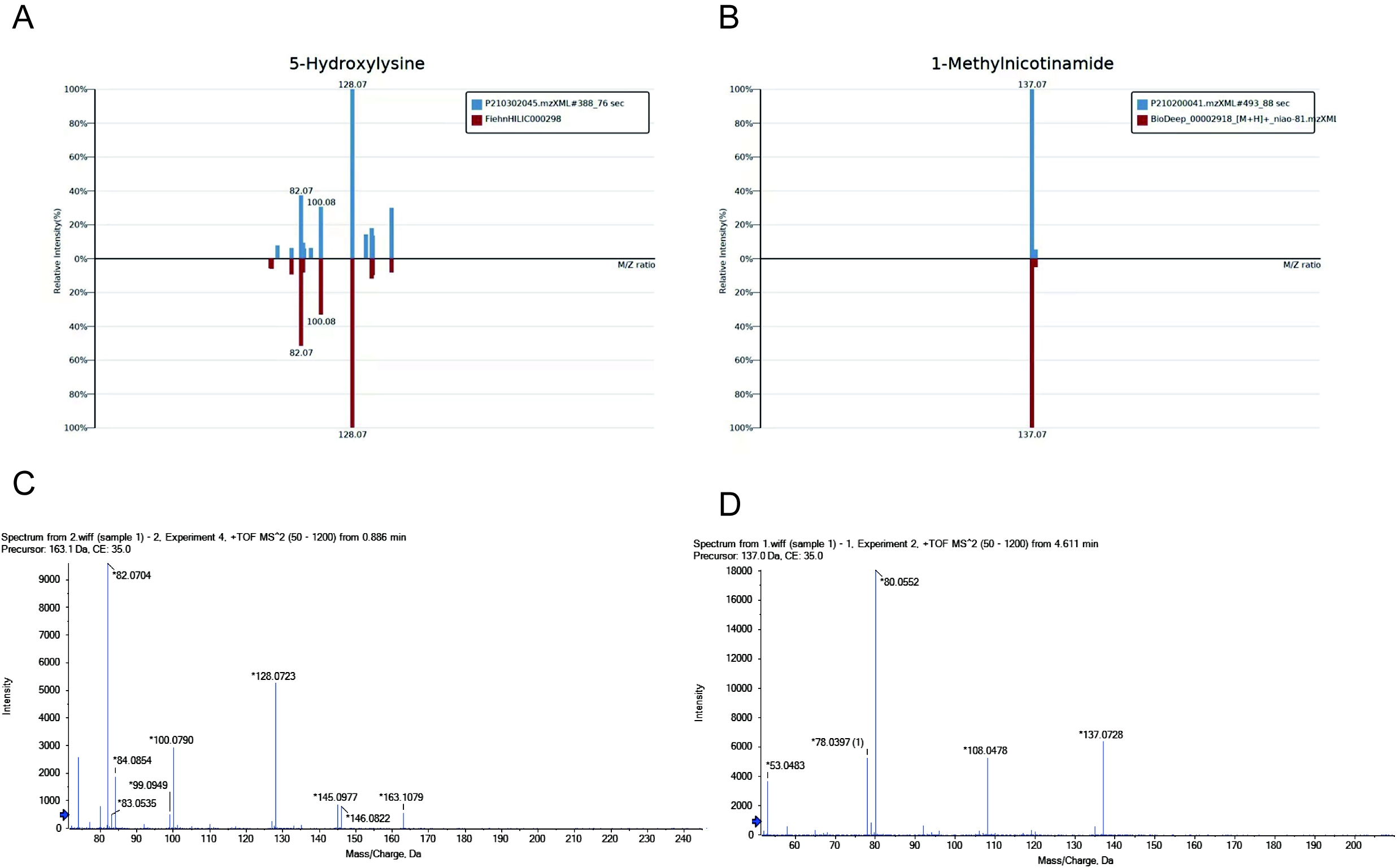

Supplement: Supplementary file 3 [file Image3.TIF]

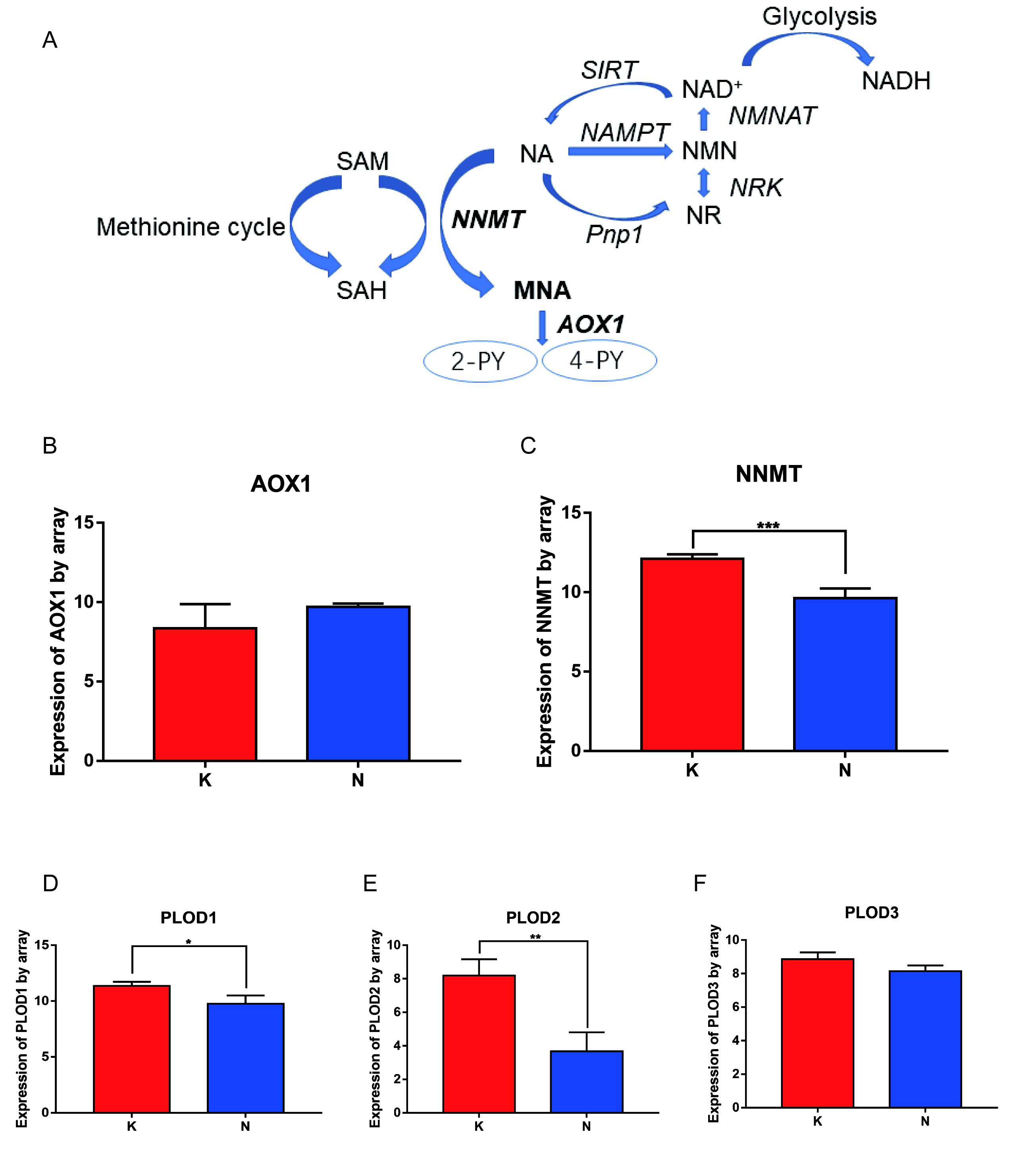

Supplement: Supplementary file 4 [file Image2.TIF]

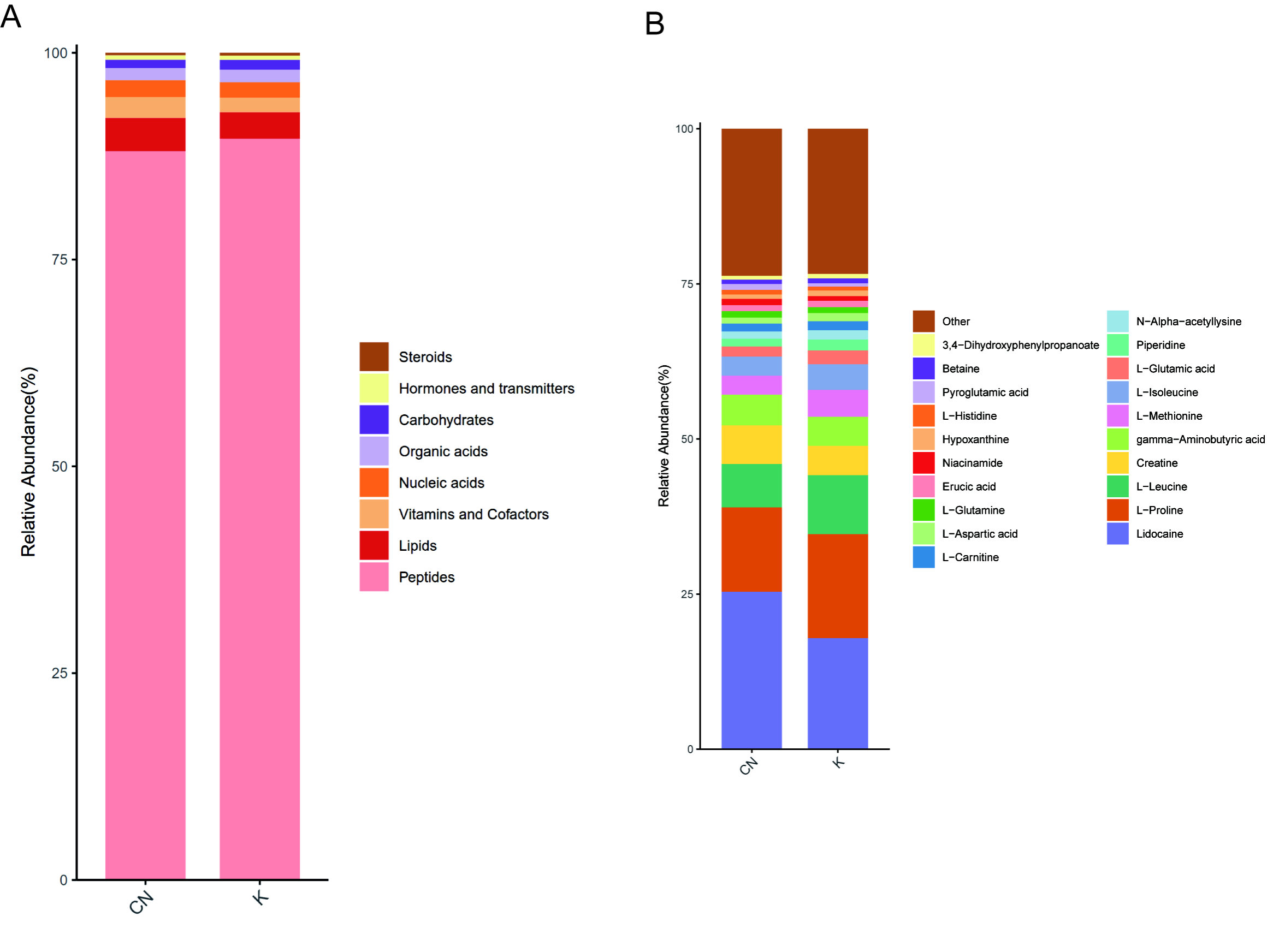

Supplement: Supplementary file 5 [file Image1.TIF]
